# Importance of Insulin Immunoassays in the Diagnosis of Factitious Hypoglycemia

**DOI:** 10.4274/jcrpe.1492

**Published:** 2014-12-05

**Authors:** Özlem Nalbantoğlu Elmas, Korcan Demir, Nusret Soylu, Nilüfer Çelik, Behzat Özkan

**Affiliations:** 1 Dr. Behçet Uz Children’s Hospital, Clinic of Pediatric Endocrinology, İzmir, Turkey; 2 Children’s Hospital, Clinic of Child and Adolescent Psychiatry, Gaziantep, Turkey; 3 Dr. Behçet Uz Children’s Hospital, Division of Biochemistry, İzmir, Turkey

**Keywords:** type 1 diabetes mellitus, DIDMOAD, factitious hypoglycemia, recurrent hypoglycemia, insulin assay

## Abstract

We report two cases emphasizing the importance of insulin assays for evaluation of hypoglycemia in diabetic patients. Case 1 was a 96/12-year-old female patient with type 1 diabetes mellitus and case 2 was a 1010/12-year-old male patient with DIDMOAD. Both patients were on a basal-bolus insulin regimen. Both were admitted because of persistent hypoglycemia. Analyses of serum samples obtained at the time of hypoglycemia initially showed low insulin and C-peptide levels. Recurrent episodes of unexplained hypoglycemia necessitated measurement of insulin levels by using different insulin assays, which revealed hyperinsulinemic hypoglycemia with low C-peptide levels, findings which confirmed a diagnosis of factitious hypoglycemia. Surreptitious administration of insulin should not be excluded in diabetic patients with hypoglycemia without taking into account the rate of cross-reactivity of insulin analogues with the insulin assay used.

## INTRODUCTION

Hypoglycemia is one of the main and dangerous complications of diabetes mellitus (DM) which may cause permanent brain damage and which may also lead to death (1). The causes of hypoglycemia in patients with diabetes are numerous (2). Incompliance with diet and inadequate glucose monitoring, organic causes (autonomic neuropathy, malabsorption, primary adrenal failure, hypopituitarism, gluten-sensitive enteropathy, Addison’s disease) and psychological issues (depression, malingering, factitious disorders) are among possible underlying causes that can be detected in the majority of the cases, but only after a challenging period of careful evaluation (3,4,5,6). Although factitious insulin use is one of the most important causes in the differential diagnosis of hypoglycemia in diabetic patients, it frequently remains overlooked (7).

Insulin is a polypeptide hormone which is secreted by the pancreas and regulates the carbohydrate metabolism. Recombinant insulin analogues are produced by modification of the human insulin molecule in order to achieve therapeutic benefits. While rapid-acting insulin analogues [insulin aspart (Novorapid^®^), insulin lispro (Humalog^®^), insulin glulisine (Apidra^®^)] mimic postprandial insulin secretion, long-acting analogues [(insulin detemir (Levemir^®^)), insulin glargine (Lantus^®^)] mimic basal insulin secretion. The ability of commercial assays to detect the serum levels of synthetic insulin analogues is variable due to different cross-reactivity, leading to diagnostic problems (8).

Here, we present two cases: the first with type 1 DM (T1DM) and the second with DIDMOAD syndrome (diabetes insipidus, DM, optic atrophy, deafness). In both patients, factitious insulin administration could be demonstrated only after use of appropriate insulin assay.

## CASE REPORTS

**Case 1**

A 9^6/12^-year-old female patient with poorly controlled T1DM was admitted due to persistent hypoglycemia in the past 10 days. The diabetes had been diagnosed 1.5 years ago. She reportedly had large fluctuations in blood glucose levels in the past one year which, however, did not necessitate hospitalization. She was on a basal-bolus insulin regimen (detemir insulin 7 units/day and aspart insulin 3-5 units thrice a day; total insulin dose, 0.66 units/kg/day), but the treatment had been withheld in the past week by the parents due to hypoglycemia. The patient’s past medical history was unremarkable. There was no history of drug use, diarrhea, steatorrhea or abdominal distention. Physical examination revealed a prepubertal female patient. Body weight was 29 kg [standard deviation (SD) score -0.2], height 130.7 cm (SD score -0.59) and body mass index was 17 kg/m^2^ (SD score 0.2). Systemic findings were normal except for presence of hepatomegaly of 3 cm and lipohypertrophy at the insulin injection sites.

Laboratory findings revealed a serum glucose level of 44 mg/dL (hypoglycemia, <50 mg/dL), alanine aminotransferase 30 IU/L (normal, 5-40), aspartate aminotransferase 28 IU/L (normal, 5-40), hemoglobin A1c 10.7% (normal, 4.8-6), free thyroxine 1.12 ng/dL (normal, 0.8-2.3) and a thyroid stimulating hormone level of 3.11 mIU/L (normal, 0.35-4.6). The urine was negative for glucose and for ketone bodies. Serum samples obtained at the time of hypoglycemia showed low insulin (0.902 uIU/mL) and C-peptide levels (0.1 ng/mL). Additionally, cortisol (24.28 µg/dL) and growth hormone (8.39 mIU/L) levels were not consistent with counter-regulatory hormone deficiency. Anti-tissue transglutaminase and anti-gliadin antibodies were negative. Hypoglycemia did not recur during the three days of observation in the hospital and she was discharged following diabetes education and an outpatient visit scheduled.

Forty days later, during which an episode of diabetic ketoacidosis developed as well, the patient was admitted with persistent hypoglycemia. Serum samples obtained at the time of hypoglycemia were measured by two different commercial insulin assays with different sensitivity for insulin analogues. While the insulin level was determined as 0.393 uIU/mL in our measurements (using Elecsys, Roche Diagnostics), it was reported to be 4160 uIU/mL (normal, <2 uIU/mL) by Architect (Abbott Laboratories, Abbott Park, Illinois) in another center. During psychiatric assessment, the patient confessed factitious insulin administration with a motivation to become and remain ill. It was also understood that the parents had left the whole responsibility of insulin administration and capillary blood glucose measurements to the patient.

**Case 2**

A 10^10/12^-year-old male patient with DIDMOAD was admitted due to recurrent episodes of hypoglycemia in the past one month. He was receiving a basal-bolus insulin regimen (glargine insulin 14 units/day and aspart insulin 8 units thrice a day; total insulin dose, 1.03 units/kg/day). The patient’s medical history was otherwise unremarkable. Both the family and the child stated that the patient had not received any additional insulin or any other medication.

His weight was 36 kg (SD score 0.23) and height was 134.5 cm (SD score -1.17). Physical examination revealed no pathological findings. Laboratory findings on admission were as follows: serum glucose 33 mg/dL (hypoglycemia, <50mg/dL), alanine aminotransferase 20 IU/L (normal, 5-40), aspartate aminotransferase 18 IU/L (normal, 5-40), hemoglobin A1c 6.83% (normal, 4.8-6). There was no glycosuria or ketonuria. At the time of hypoglycemia, insulin and C-peptide levels were not high: 4.3 uIU/mL and 0.1 ng/mL, respectively. Anti-tissue transglutaminase and anti-gliadin antibodies were negative. Cortisol (21.53 µg/dL) and adrenocorticotropic hormone (18 pg/mL) levels were inconsistent with adrenal insufficiency. However, hypoglycemia was persistent. Since we found out that insulin level as measured by Roche Elecsys/E170 insulin assay (Roche Diagnostics, Indianapolis, Indiana) was not capable of detecting analogues, the analysis from the same sample was repeated by using Advia Centaur (Siemens Healthcare Diagnostics, Tarrytown, New York). Serum insulin level turned out to be very high (>300 uIU/mL), while the C-peptide was low, suggesting factitious hypoglycemia. The patient was then referred to a psychiatric clinic.

## DISCUSSION

Cases of factitious hypoglycemia due to surreptitious administration of insulin as well as of sulfonamides have been reported (9,10,11). Insulin was misused by patients for purposes such as catching attention or skipping school (11,12). Reaching a state of hypoglycemia to be able to eat sugar-containing foods or a state of euphoria due to hypoglycemia were other cited reasons (11,12,13,14). Insulin has also been used to commit suicide (13,14,15,16). There are also reports of self-inflicted cases of hypoglycemia due to frequent use of prime function on an insulin pump, which provides small insulin doses to clean the air within the tube and are not recorded in daily insulin history (11,12).

The insulin analogues are modified forms of the human insulin molecule in order to enhance therapeutic effectiveness. Human insulin is composed of 2 polypeptide chains (A chain and B chain) which are stabilized by disulfide bonds. Although this 2-chain structure is maintained in the insulin analogues, the chains contain a variety of modifications (15). The terminus of the B-chain and the immunodominant A-chain are the antigenic determinants of human insulin (15,16). The short-acting insulin analogue insulin lispro undergoes a change by the switch of the proline and lysine residues at B28 and B29. Insulin aspart was created by a single substitution of aspartic acid for proline at position B28. The long-acting insulin analogue glargine is altered by exchange of the A21 asparagine by a glycine and it contains two additional arginines at the C-terminus of the B-chain. In insulin glulisine, the natural sequence of asparagine at position B3 and lysine at position B29 are substituted by lysine and glutamic acid, respectively (17,18). Insulin detemir differs from human insulin in that the amino acid threonine in position B30 has been omitted and a C14 fatty acid chain has been attached to the amino acid B29. Due to these, insulin analogues show different immunoreactivity profiles compared to human insulin.

Both of our cases were admitted with persistent hypoglycemia. Initial measurements disclosed low insulin levels in both cases. In addition to the relevant data presented by Neal and Han in 2008, recent literature regarding cross-reactivity rates of the insulin assays for glulisine and detemir are shown in Table 1 (10). Roche Elecsys/E170 insulin assay, which was the first method used in both of our cases, is an electrochemiluminescence immunoassay and uses a biotinylated anti-insulin antibody and a monoclonal anti-insulin antibody labeled with an electrochemiluminescent ruthenium complex to detect human insulin levels. However, the assay demonstrates very low cross-reactivity (<0.02%) for insulin aspart, insulin lispro and glargine (19,20). Merrigan et al (21) reported that the E170 assay does not detect insulin glulisine and detemir as well. However, it was reported that M1 metabolite (21A-Gly-insulin) of glargine could be detected by E170 assay (22).

In our two patients, demonstration of factitious hypoglycemia was accomplished by detection of high insulin levels using Architect and Advia Centaur insulin assays, which are electrochemiluminescence immunoassays as well. Both assays use same types of antibodies to human insulin: a monoclonal mouse anti-insulin antibody labeled with acridinium ester and a monoclonal mouse anti-insulin antibody covalently coupled to paramagnetic particles. The cross-reactivity rates of the Advia Centaur for insulin lispro, aspart and glargine are 90%, 126% and 143%, respectively (8). Insulin aspart showed 75% cross-reactivity on the Architect insulin assay, while insulin lispro and glargine had 100% and 83% cross-reactivity, respectively (22).

In summary, surreptitious administration of insulin should be considered as a possible scenario in diabetic patients with hypoglycemia. However, as experienced in these cases, variability in detection of insulin analogues by different insulin assays should be taken into account in the diagnosis.

## Figures and Tables

**Table 1:**
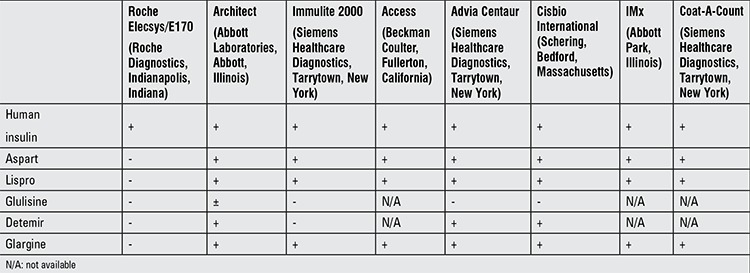
Comparison of commercial insulin assays (8,10,16,17,19,21,22)

## References

[1] MacLeod KM, Hepburn DA, Frier BM (1993). Frequency and morbidity of severe hypoglycemia in insulin-treated diabetic patients. Diabet Med.

[2] Tattersall RB, Frier B, Fisher M (1993). Frequency and causes of hypoglycaemia. Hypoglycaemia and Diabetes.

[3] Tattersall RB (1997). Brittle diabetes revisited: the Third Arnold Bloom Memorial Lecture. Diabet Med.

[4] Eliaschewitz FG, Franco DR (2009). [Does brittle diabetes exist as an entity?]. Arq Bras Endocrinol Metabol.

[5] Hardy KJ, Burge MR, Boyle PJ, Scarpello JH (1994). A treatable cause of recurrent severe hypoglycemia. Diabetes Care.

[6] Vantyghem MC, Press M (2006). Management strategies for brittle diabetes. Ann Endocrinol (Paris).

[7] Hess C, Thomas A, Thevis M, Stratmann B, Quester W, Tschoepe D, Madea B, Musshoff F (2012). Simultaneous determination and validated quantification of human insulin and its synthetic analogues in human blood serum by immunoaffinity purification and liquid chromatography-mass spectrometry. Anal Bioanal Chem.

[8] Owen WE, Roberts WL (2004). Cross-reactivity of three recombinant insulin analogs with five commercial insulin immunoassays. Clin Chem.

[9] Vanelli M (2002). Munchausen’s syndrome by proxy web-mediated in a child with factitious hyperglycemia. J Pediatr.

[10] Neal JM, Han W (2008). Insulin immunoassays in the detection of insulin analogues in factitious hypoglycemia. Endocr Pract.

[11] Osipoff JN, Sattar N, Garcia M, Wilson TA (2010). Prime-time hypoglycemia: factitious hypoglycemia during insulin-pump therapy. Pediatrics.

[12] Franklin VL, Bluff S, Ramsay V, Sturrock C, Greene SA, Alexander V (2007). Unexplained hypoglycaemia on a pump. Pediatr Diabetes.

[13] Kaminer Y, Robbins DR (1989). Insulin misuse: a review of an overlooked psychiatric problem. Psychosomatics.

[14] Cassidy EM, O’Halloran DJ, Barry S (1999). Insulin as a substance of misuse in a patient with insulin dependent diabetes mellitus. BMJ.

[15] Hirsch IB (2005). Insulin analogues. N Engl J Med.

[16] Heald AH, Bhattacharya B, Cooper H, Ullah A, McCulloch A, Smellie S, Wark G (2006). Most commercial insulin assays fail to detect recombinant insulin analogues. Ann Clin Biochem.

[17] Becker RH, Frick AD (2008). Clinical pharmacokinetics and pharmacodynamics of insulin glulisine. Clin Pharmacokinet.

[18] Becker RH (2007). Insulin glulisine complementing basal insulins: a review of structure and activity. Diabetes Technol Ther.

[19] Sapin R, Le Galudec V, Gasser F, Pinget M, Grucker D (2001). Elecsys insulin assay: free insulin determination and the absence of crossreactivity with insulin lispro. Clin Chem.

[20] Ottesen JL, Nilsson P, Jami J, Weilguny D, Dührkop M, Bucchini D, Havelund S, Fogh JM (1994). The potential immunogenicity of human insulin and insulin analogues evaluated in a transgenic mouse model. Diabetologia.

[21] Merrigan SD, Owen WE, La`ulu SL, Wyness SR, Roberts WL, Straseski JA (2012). Cross-Reactivity of Modern Insulin Analogs Insulin Detemir and Insulin Glulisine With Six Automated Insulin Immunoassays. 51th Annual European Society For Paediatric Endocrinology Meeting.

[22] Moriyama M, Hayashi N, Ohyabu C, Mukai M, Kawano S, Kumagai S (2006). Performance evaluation and cross-reactivity from insulin analogs with the ARCHITECT insulin assay. Clin Chem.

